# Biochemical and phylogenetic characterization of the wastewater tolerant *Chlamydomonas biconvexa* Embrapa|LBA40 strain cultivated in palm oil mill effluent

**DOI:** 10.1371/journal.pone.0249089

**Published:** 2021-04-07

**Authors:** Patrícia Verdugo Pascoal, Dágon Manoel Ribeiro, Carolina Ribeiro Cereijo, Hugo Santana, Rodrigo Carvalho Nascimento, Andrei Stecca Steindorf, Lorena C. G. Calsing, Eduardo Fernandes Formighieri, Bruno S. A. F. Brasil

**Affiliations:** 1 Embrapa Agroenergia, Brasília, Distrito Federal, Brazil; 2 Universidade Federal da Bahia, Salvador, Bahia, Brazil; 3 Universidade de Brasília, Brasília, Distrito Federal, Brazil; 4 Universidade Zambeze, Sofala, Mozambique; 5 Universidade Federal do Tocantins, Gurupi, Tocantins, Brazil; Bharathidasan University, INDIA

## Abstract

The increasing demand for water, food and energy poses challenges for the world´s sustainability. Tropical palm oil is currently the major source of vegetable oil worldwide with a production that exceeds 55 million tons per year, while generating over 200 million tons of palm oil mill effluent (POME). It could potentially be used as a substrate for production of microalgal biomass though. In this study, the microalgal strain *Chlamydomonas biconvexa* Embrapa|LBA40, originally isolated from a sugarcane vinasse stabilization pond, was selected among 17 strains tested for growth in POME retrieved from anaerobic ponds of a palm oil industrial plant located within the Amazon rainforest region. During cultivation in POME, *C*. *biconvexa* Embrapa|LBA40 biomass productivity reached 190.60 mgDW • L^-1^ • d^-1^ using 15L airlift flat plate photobioreactors. Carbohydrates comprised the major fraction of algal biomass (31.96%), while the lipidic fraction reached up to 11.3% of dry mass. Reductions of 99% in ammonium and nitrite, as well as 98% reduction in phosphate present in POME were detected after 5 days of algal cultivation. This suggests that the aerobic pond stage, usually used in palm oil industrial plants to reduce POME inorganic load, could be substituted by high rate photobioreactors, significantly reducing the time and area requirements for wastewater treatment. In addition, the complete mitochondrial genome of *C*. *biconvexa* Embrapa|LBA40 strain was sequenced, revealing a compact mitogenome, with 15.98 kb in size, a total of 14 genes, of which 9 are protein coding genes. Phylogenetic analysis confirmed the strain taxonomic status within the *Chlamydomonas* genus, opening up opportunities for future genetic modification and molecular breeding programs in these species.

## Introduction

Water, food and energy security, in a context of climatic changes, are major worldwide challenges for the 21st century. Therefore, the advancement of biofuel production and the integration of industrial processes in biorefineries will be pivotal in order to provide energy, food and fibers, such as cellulose for paper production, in a sustainable manner [1].

Brazil has one of the most advanced and well-established biofuel policies among BRICS nations and the developed economies of the world [2,3]. Since the adoption of the National Fuel Alcohol Program (proálcool), in 1975, and the National Program of Production and Use of Biodiesel (PNPB), in 2004, the country has leveraged its production of biofuels and has become the second largest bioethanol and biodiesel producer in the world. The country’s light fleet is largely composed of flex-fuel vehicles and a National Biofuel Policy (RenovaBio) was established in 2017 to increase the production and use of biofuels through the commercialization of decarbonization credits [3,4].

Among the different feedstocks available for biodiesel and edible oil production, palm oil is one of the most efficient crops regarding land use and productivity in tropical regions. Worldwide palm oil production exceeds 55 million tons annually, representing a market of over $ 34 billion a year [5]. Currently, Brazil is the nineth world’s producer of palm oil concentrating the large majority of its plantations within the Amazon rainforest.

The Amazon region spans over 6.7 thousand square kilometers of tropical rainforest, comprising one of the most species-rich biodiversity hotspots in the world. In order to prevent the primary and secondary forest clearing, Brazil`s Sustainable Palm Oil Production Program, since 2010, regulates palm oil production by restricting new oil palm plantations in Amazonia, including legally protected parks, indigenous reserves and intact forest areas. As a result, it has been shown that direct conversion of intact forest to oil palm declined 4% from 2006–2010, to less than 1% from 2010–2014 [6]. However, despite these efforts to protect the forest areas, crude palm oil production can still pose as an important threat to the environment. For example, the production of 1 ton of crude palm oil requires between 5 and 7.5 tons of water, leading to the generation of large volumes of wastewater in the form of palm oil mill effluent (POME) [7]. Given the continuous increase in palm oil production within the Amazon region, POME generation at large volumes might pose a challenge for the sustainable growth of this industry.

POME is composed of 95–96% water, 0.6–0.7% residual oil and 4–5% total solids. It has high chemical and biochemical oxygen demands and contains significant concentrations of nitrogen and phosphorus that can cause severe pollution to the environment [7]. Palm oil industries typically use stabilization pond-based systems to treat POME wastewater. This type of treatment is particularly common in tropical conditions due to the high temperatures, solar radiation, presence of oxygen and phytoplankton that help stabilize the effluent [8]. The main advantages are the high efficiency for organic and solid matter removal and the low cost of implementation and maintenance. However, pond systems require large areas, long stabilization periods (usually over 3 months) and do not completely eliminate the organic and inorganic load of the wastewater [9,10].

POME can potentially be used as a cultivation medium for the production of microalgae, opening up opportunities for integrated production of high value-added algal biomass within the palm oil industry [7,11]. The use of microalgae has been increasingly attracting interest worldwide as an alternative biomass source, due to its wide biotechnological applications in food, feed, energy and material production, its flexibility in terms of cultivation conditions, as well as its sustainability and contribution to the reduction of greenhouse gas emissions [4,12]. However, low cost culture systems, improvements in nutrient use efficiency and selection of productive strains, still pose as challenges for the economic viability of large scale production [13]. In this context, the use of industrial wastewater and residues stands as a potential low cost and abundant source for the formulation of culture media for algae [14].

Previous studies have shown the viability of cultivating microalgae in different concentrations of POME at a laboratory scale [15,16] as well as the use of acid or antibiotic pretreatment to control contaminants has also been proposed [17]. However, information regarding microalgae cultivation in POME retrieved from anaerobic stabilization ponds without nutrient supplementation using closed photobioreactors and under non-axenic conditions is still scarce. Furthermore, the chemical composition of algal biomass produced in POME has not yet been characterized in detail.

Recently, the strain *Chlamydomonas biconvexa* Embrapa|LBA40 has been shown to produce high amounts of protein-rich biomass when cultivated in sugarcane vinasse, an abundant wastewater from ethanol plants, uncover a promising biotechnological potential [18,19]. *C*. *biconvexa* is a freshwater species that presents characteristic oval shaped cells with two anterior flagella. Often forms colonial aggregates of immobile nonflagellated individuals, referred as palmella stage [20]. assigned to the *Chlamydomonas* genus solely based on morphologic and physiologic analysis, since genomic and phylogenetic information about this species are virtually absent from the literature.

The main purpose of this study is to evaluate whether *Chlamydomonas biconvexa* Embrapa|LBA40 is capable of producing large amounts of value-added biomass during non-axenic cultivation in POME without nutrient supplementation, while bioremediating its nitrogen and phosphorous load. Here, we show that *C*. *biconvexa* Embrapa|LBA40 strain presents the highest microalgae productivity reported to date when grown in airlift flat plate photobioreactors using non-supplemented POME retrieved from anaerobic ponds as cultivation medium. The strain was also efficient in wastewater bioremediation, potentially reducing the area used for POME stabilization and contributing to sustainable palm oil production within the Amazon region. Biochemical characterization of proteins, carbohydrates and lipids fractions within algae biomass was performed in order to evaluate algae bioproducts production potential in a biorefinery approach. Furthermore, the complete mitochondrial genome sequence and phylogenetic analysis of *C*. *biconvexa* Embrapa|LBA40 is presented.

## Materials and methods

### Microalgal strains

Seventeen (17) microalgae strains from the Collection of Microorganisms and Microalgae for Agroenergy and Biorefineries of the Brazilian Agricultural Research Corporation—Embrapa (Brasília-DF) [19,21] were used in this study (S1 Table). Microalgae strains were maintained in erlenmeyer flasks containing 150 mL working volume of bold basal medium (BBM) at 26 ± 1°C, with aeration of 5 L·h^-1^of atmospheric air, light intensity of 50 μEm^-2^ s^-1^ (3750 lux) at and 12/12h light/dark cycle.

### Palm oil mill effluent

**Fig 1:**
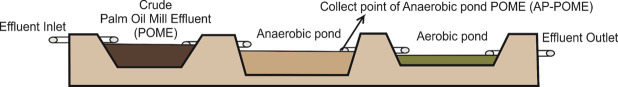
Representation of Palm Oil Mill Effluent (POME) wastewater treatment ponds (DENPASA—Dendê do Pará S/A).

The palm oil mill effluent used in this study was collected at the exit of the POME anaerobic stabilization pond at a palm oil industrial plant (Dendê do Pará S/A) located within the Brazilian Amazonian region at the Pará state (Fig 1). It is referred hereafter as “anaerobic pond POME”, or simply, “AP-POME”. The treatment system used in this industrial plant is composed of two sequential ponds, one anaerobic and the second aerobic (Fig 1). Prior to experimentation, AP-POME was prepared by centrifugation at 4800 ×g for 10 minutes to remove suspended solids. This step resulted in a decrease in AP-POME turbidity from 396 NTU prior to centrifugation to 18 NTU after. No alteration in the color of AP-POME was detected. AP-POME was stored at 4°C until use.

### Microalgae screening for growth in anaerobic pond palm oil mill effluent

Screening was carried out in 500 ml erlenmeyer flasks containing 250 ml of AP-POME as culture medium. Batch culturing was independently conducted in triplicates for each of the 17 strains at a constant aeration of 5 L·h^-1^of atmospheric air, at 26 ± 1° C, light intensity of 100 μEm^-2^ s^-1^ (7500 lux), and 12/12 h light/dark cycle. Microalgae growth was monitored through periodic measures of absorbance at 680 nm and microscopic inspection of culture samples during 10 days of cultivation. The absorbance of AP-POME prior to algal inoculation was used as blank and the basal growth rate (*μ*) was calculated according to the equation μ = [(OD_2_ –OD_AP-POME_)/(OD_1_ –OD_AP-POME_)]/*t*_2_−*t*_1._

### *Chlamydomonas biconvexa* Embrapa|LBA40 strain cultivation

*Chlamydomonas biconvexa* Embrapa|LBA40 strain was cultivated using 250 mL of AP-POME or BBM (control) under different conditions: i) axenic culture using BBM at 12/12 h light/dark cycles; ii) axenic culture using AP-POME at 12/12 h light/dark cycles; iii) axenic culture using AP-POME in the dark; iv) non-axenic culture using AP-POME at 12/12 h light/dark cycles; v) non-axenic culturing using 50% AP-POME diluted in distillate water at 12/12 h light/dark cycles. For axenic cultures experiments, BBM or AP-POME were autoclaved at 121° C for 15 minutes and subsequently stored at 4°C until use. Batch cultures were conducted in triplicates under aeration of 5 L·h^-1^of atmospheric air, at 26°C ± 1°C, at light intensity of 100 μEm^-2^ s^-1^(7500 lux) (when applicable). Algae growth kinetics was evaluated through cell counting using a Neubauer chamber as described by Santana and collaborators [22].

### Microalgae cultivation in airlift flat plate photobioreactors

*Chlamydomonas biconvexa* Embrapa|LBA40 was batch cultivated in 15 L acrylic airlift flat plate photobioreactors under non-axenic conditions. The working volume of 13 L of cultivation medium (BBM or AP-POME) was used, with constant aeration of 60L·h^-1^ and CO_2_ supplementation adjusted to 5% of the air flow. Experimentation was conducted at 12h/12h light/dark cycling regime, at a light intensity of 495 μEm^-2^ s^-1^ (35000 lux) and temperature of 25°C during dark periods and 35°C during the light periods. Biomass dry weight (DW) was gravimetrically determined [22] using 10 ml samples retrieved from cultures from each photobioreactor replicate at the initial time and at 5 days intervals during 15 days of culturing.

### The chemical parameters of palm oil mill effluent (POME)

The chemical parameters of crude POME and AP-POME before and after 5, 10 and 15 days of microalgae cultivation using airlift flat plate photobioreactors were determined. Samples were collected from each photobioreactor replicate, centrifuged for 10 min at 4800 ×g and the supernatants were used for analysis. The physicochemical characteristic and methods used were: biochemical oxygen demand (BOD)—SM5210B; chemical oxygen demand (COD)—QAM.IT.FQ.16A; total organic carbon—SM 4500-O/D; nitrate (NO_3_^-^)—ABNT NBR 12620:1992; nitrite (NO_2_^-^)—SM 4500-NO2-B; ammoniacal nitrogen (NH_4_^+^)—SM 4500-NH3 F; phosphate (PO_4_^3-^)—SM 4500-P E and total potassium (K^+^)—SM 3500-K B [22].

### Composition of algal biomass

After cultivation, algal biomass was harvested by centrifugation for 10 min at 4800 × g and then freeze dried. Total protein content was measured by the quantification of nitrogen using micro-Kjeldahl with the microalgae-specific conversion factor of 4.78 [23]. Carbohydrate content, fractions, total solids and ashes were quantified following the protocol described by Van Wychen and Laurens [24].

The lipid content and fatty acid profiles were determined according to Van Wychen et al. [25]. Briefly, the lipid fraction was obtained by ether extraction (EE) with petroleum ether at 90°C for 90 minutes (Ankom XT15). Experimentation was carried out in triplicates using 100 mg of freeze-dried biomass. Samples were placed in hydrophobic filter bags (XT4 Filter Bags, ANKOM Technology), with porosity of 3 microns that only allows the extraction of nonpolar compost (lipid fraction) soluble in petroleum ether, the quantification is calculated by the gravimetric difference of the biomass after the extraction.

Fatty acid profiles were determined through gas chromatography after treatment of 10 mg of biomass for transesterification with 0.2 ml of chloroform: methanol (2:1, v/v) and 0.3 ml of 0.6 M HCl in methanol heated at 85°C in dryi-block for 1 hour. After heating, 1 ml hexane was used to extract the Fatty acid methyl esters (FAMES). FAMEs were separated by gas chromatography using an Agilent 7890 A gas chromatograph (Agilent Technologies, California, USA), coupled with a flame ionization detector (FID) and a fused silica capillary column (100 m x 250 μm x 0.2 μm, Supelco SP). The operating parameters were set as follows: detector temperature, 260°C column temperature, 140°C for 5 minutes, programmed to increase 4°C/min, up to 240°C, with a final running time of 48 minutes. The carrier gas was Helium at 1.2 mL•min-1, with injection of 1 μL sample. The retention times of fatty acids were compared to those of standard methylesters (Sigma-Aldrich, St. Louis, MO, USA). Retention times and percentages of the peak area were calculated automatically by the ChemStation Software. Fatty acids quantifications (FA) were performed using the methyl ester of nonadecanoate acid (Sigma-Aldrich, USA) as an internal standard.

### Statistical analysis

Experiments were conducted using biological triplicates (n = 3). Algal biomass productivity (*mgDW* ∙ *L*^−1^ ∙ *day*^−1^) was calculated using the equation described by Kishi and Toda [26]. POME and algal Biomass composition data were subjected to analysis of variance (ANOVA) at 5% probability followed by a Tukey test, using the software Action Stat version 3.5.

### Genomic analysis

#### Sequencing

Sixty milligrams of *C biconvexa* Embrapa|LBA40 fresh biomass were used for genomic DNA extraction following the protocol previously described by Doyle [27]. The genomic DNA was further fragmented for the: construction of two libraries shotgun library (2 x 250 bp), sequenced on the Illumina MiSeq platform; and jump library 3 kb (2 x 125 bp), sequenced on the Illumina HiSeq 2500 platform according to the manufacturer’s protocols. Subsequently, *in silico* analyses were performed at the Laboratory of Bioinformatics in Bioenergy (LBB) of Embrapa Agroenergy (Brasília, Brazil).

The FastQC tool [28] was used to evaluate the quality of the sequence data set. The adapters were trimmed using the Trimmomatic software [29] with the following adapted parameters: ILLUMINACLIP:TruSeq3-PE.fa:2:30:10 SLIDINGWINDOW:4:20 LEADING:15 TRAILING:10 MINLEN:30.

### Mitochondrial genome assembly and annotation

*De novo* genome assembly was performed with whole genome shotgun assembler ALLPATHS-LG v.3 [29], using low paired-end read coverage (2X).The annotation process was accomplished using MITOS2 Web Server [30], Reference: 63 –Opisthokonta and Genetic code: 16 Chlorophycean. The visualization of mitogenome annotation was performed with OGDRAW [31], using the *Chlamydomonas reinhardtii* mitogenome as a model, available at GEO (NCBI’s Gene Expression Omnibus) under accession number GSE101944 [32]. The *C*. *biconvexa* Embrapa|LBA40 mitogenome sequence data was deposited at NCBI GenBank under the accession number MG916975.1.

### Phylogenetic tree construction

Phylogenetic analysis was performed using the mitochondrial cox1 (cytochrome oxidase subunit 1) subunit gene as a barcode. The cox1 protein annotated sequence of *C*. *biconvexa* Embrapa|LBA40 mtDNA was concatenated to other twenty-nine green algae cox 1 protein sequences obtained from GenBank. Phylogenetic tree construction was conducted in MEGA X [33]. The sequences were aligned by ClustalW and Maximum Likelihood method and JTT matrix-based model was used to evolutionary inference. The bootstrap consensus tree inferred from 100 replicates. The percentage of replicate trees in which the associated taxa clustered together in the bootstrap test (100 replicates) are shown next to the branches. This analysis involved 30 amino acid sequences. There were a total of 541 positions in the final dataset.

## Results and discussion

### Screening of microalgae strains for growth in anaerobic pond palm oil mill effluent

**Fig 2:**
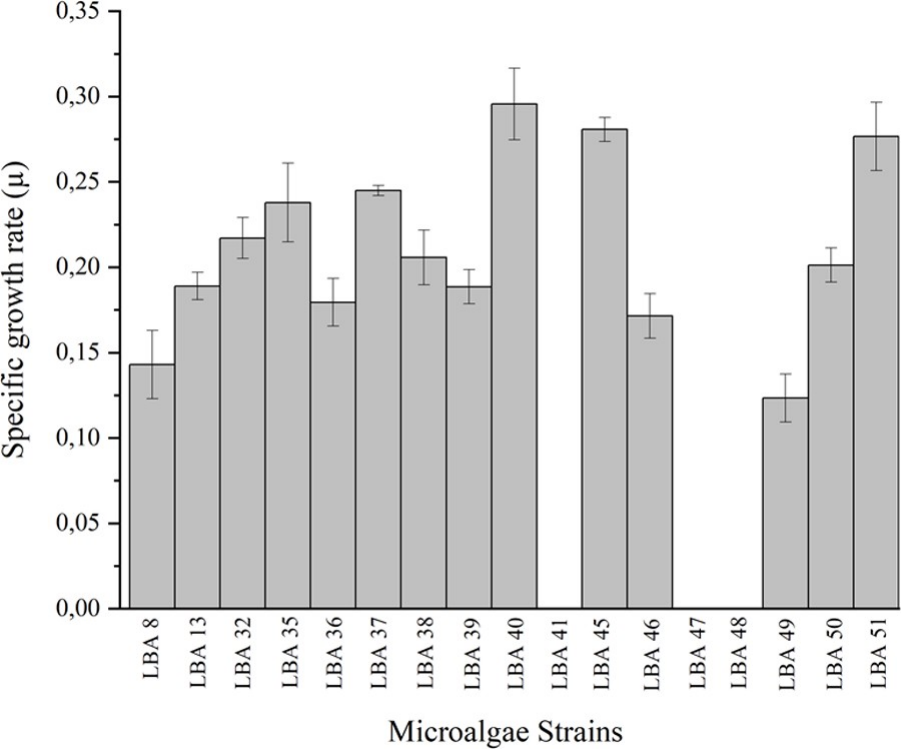
Screening of microalgae strains for growth in anaerobic pond palm oil mill effluent (AP-POME): Screening was carried out in 500 ml erlenmeyer flasks containing 250 ml of AP-POME as culture medium. Batch culturing was independently conducted in triplicates for each of the 17 strains at a constant aeration of 5 L·h^-1^of atmospheric air, at 26 ± 1°C, light intensity of 100 μEm^-2^ s^-1^ (7500 lux), and 12/12h light/dark cycle. Microalgal growth was monitored through periodic measures of absorbance at 680nm and microscopic inspection of culture samples during 10 days of cultivation. The absorbance of AP-POME prior to algal inoculation was used as blank. Initial and final absorbances were used to calculate basal growth rate (*μ*). *Results are presented as mean ± error bars of triplicate experiments (n = 3).

The selection of robust strains adapted for wastewater growth and with high biomass productivity is a crucial step for the viability of the cultivation system [1,22]. Here, seventeen (17) strains of microalgae (S1 Table) were screened for growth using AP-POME as substrate (Fig 2).

The strains used were originally collected from distinct neotropical biomes, including not only natural environments but also anthropizedones, such as rural wastewater and sugarcane vinasse stabilization ponds [19]. The strains *Chlamydomonas biconvexa* Embrapa|LBA40, *Chloromonas* sp. Embrapa|LBA45 and *Chlorococcum* sp. Embrapa|LBA51 presented the highest specific growth rates among the microalgae strains tested. On the other hand, the strains *Chlamydomonas* sp. Embrapa|LBA41, *Chlorococcum* sp. Embrapa|LBA47 and *Chlorococcum* sp. Embrapa|LBA48 did not present any growth (Fig 2). The growth rate differences observed among closely related strains (i.e.: *C*. *biconvexa* Embrapa|LBA40 and *Chlamydomonas* sp. Embrapa|LBA41; *Chlorococcum* sp. Embrapa|LBA51 and *Chlorococcum* sp. Embrapa|LBA48) indicates that tolerance to POME might be strain-specific.

*C*. *biconvexa* Embrapa|LBA40 strain has been recently shown to produce high amounts of protein-rich biomass when cultivated in sugarcane vinasse wastewater, either diluted 50% in water or chemically clarified [19,22]. The capability of achieving high growth rates in two distinct industrial wastewaters (i.e.: sugarcane vinasse and POME) suggests that *C*. *biconvexa* Embrapa|LBA40 has an unusual versatility to tolerate adverse growth conditions and might have potential for large scale production in a biorefinery context. Therefore, this strain was selected for further biochemical and phylogenetic characterization.

### Algal growth in anaerobic pond palm oil mill effluent

**Fig 3:**
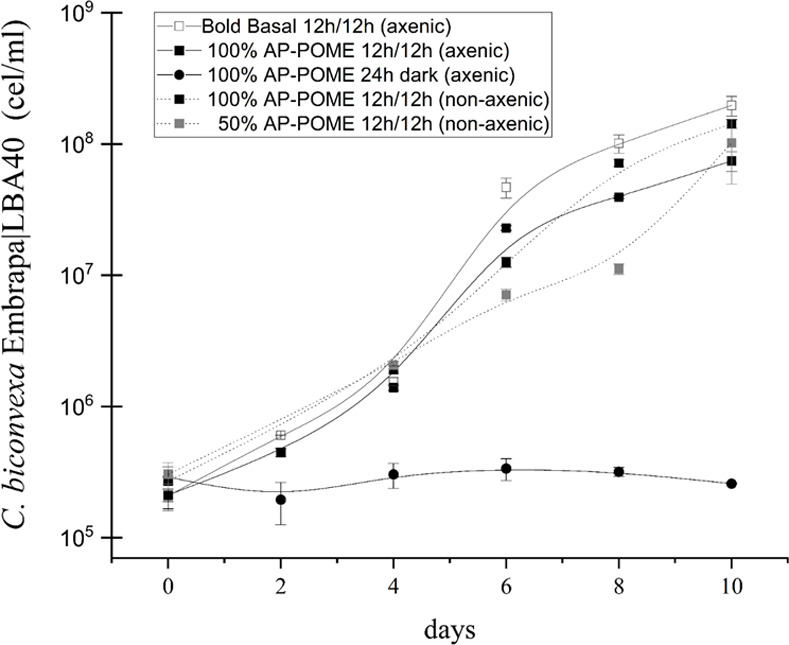
Growth dynamics of *Chlamydomonas biconvexa* Embrapa|LBA40 under different conditions. The strain was cultivated in erlenmeyer flasks using 250 mL of AP-POME or BBM (control) under different conditions: Axenic culture using BBM and 12/12 h light/dark cycles (−□−), axenic culture using undiluted AP-POME in the light (−■−); axenic culture using undiluted AP-POME in the dark (−●−), non-axenic culture using undiluted AP-POME and 12/12 h light/dark cycles (··■··), non-axenic culturing using 50% diluted AP-POME in distillate water and 12/12 h light/dark cycles (··■··). Batch cultures were aerated with 5 L·h^-1^ of atmospheric air, at 26°C ± 1°C, at light intensity of 100 μEm^-2^ s^-1^ (7500 lux) (when applicable). The growth kinetics of the algae was evaluated through cell counting using a Neubauer chamber. Results shown are the mean of biological triplicates of the experiment (n = 3).

*C*. *biconvexa* Embrapa|LBA40 strain was grown in aerated erlenmeyer flasks containing AP-POME under different conditions in order to characterize the effect of light and microbial contamination upon algal growth (Fig 3).

No algal growth was detected in the absence of light. On the other hand, *C*. *biconvexa* Embrapa|LBA40 presented similar growth patterns either in 100% and 50% non-axenic cultures performed under light/dark cycles using both Bold´s Basal Medium (BBM) and AP-POME, indicating robust photo-dependent algae growth albeit the presence of microbial competitors (Fig 3). Similarly, *C*. *biconvexa* Embrapa|LBA40 has been reported to perform light-dependent growth when cultivated in sugarcane vinasse [14].

**Fig 4:**
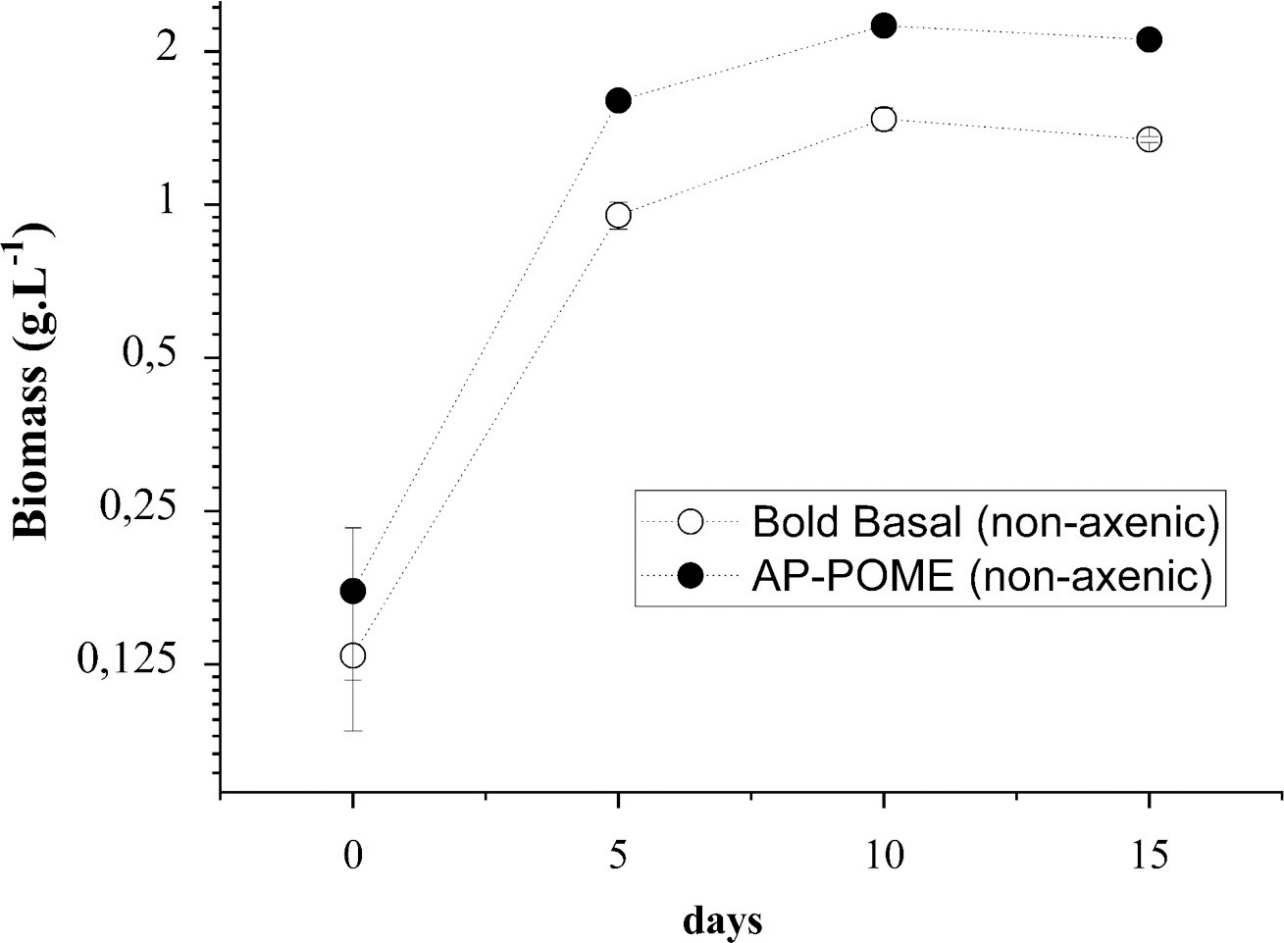
Growth dynamics of *Chlamydomonas biconvexa*Embrapa|LBA40 in airlift flat plate photobioreactors. The strain was batch cultivated using in airlift flat plate photobioreactors under non-axenic conditions. The working volume of 13 L of cultivation medium, bold basal medium (BBM) (··○··) or anaerobic pond palm oil mill effluent (AP-POME) (··●··), was used with constant aeration of 60 L·h-1 and CO2 supplementation adjusted to 5% of the air flow. Experimentation was conducted at 12h/12h light/dark cycling regimen, at a light intensity of 495 μEm^-2^ s^-1^ (35000 Lux) and temperature of 25° C during dark periods and 35° C during the light periods. Biomass dry weight (DW) was gravimetrically determined using 10 ml samples retrieved from cultures from each photobioreactor replicate at the initial time and at 5 days intervals during 15 days of culturing. Results shown are the mean of biological triplicates of the experiment (n = 3).

Cultivation of *C*. *biconvexa* Embrapa|LBA40 in AP-POME was further scaled-up using 15L capacity airlift flat plate photobioreactors, under constant aeration with atmospheric air enriched with 5% CO_2_ (Fig 4). As shown on Fig 4, the exponential growth phase of the algae occurs until the 5th day of cultivation, followed by a phase of declining relative growth / stationary phase from the 5th to the 10th day of cultivation. Fig 4 also indicates that cultures are probably entering the death phase after the 10^th^ day, which is characterized by a slight decrease in algal biomass until the 15^th^.

Culture volume expansion testing is an essential step for large scale application, and the flat plate geometry represents one of the major types of photobioreactors available for production of microalgae [18]. After five days of cultivation, *Chlamydomonas biconvexa* presented productivities of 190,6mgDW • L^-1^ • d^-1^ in POME (Fig 4 and Table 3). This is the highest algae biomass productivity reported to date using an aerobic pond derived POME without nutrient supplementation (Table 1).

**Table 1:** Comparison of biomass productivities and nutrient removal efficiencies obtained with microalgae species cultured in palm oil mill effluent (POME) under various conditions.

Strain	Culture conditions	Biomass Productivity (mg.L^-1^.d^-1^)	Nutrient removal	Reference
***Chlamydomonas biconvexa*** **Embrapa|LBA40**	100% POME from anaerobic pond, supplemented with 5% of CO2 in 15L flat plate airlift photobioreactor	190.60	34.70% of Nitrate 99% of Nitrite 99% of Ammonium	This study
***Chlorella*** **sp.** ***(UKM2)***	100%POME from anaerobic pond supplemented with 10% of CO2 in 2 L flask.	64	80.90% of TN	[10]
***Scenedesmus*** **sp.**	84% diluted POME from anaerobic pond in 1L flask	20.40	74.40% of COD 37% of TN 52.42% of Phosphate	[16]
***Chlorella sorokiniana*** **C 212**	75% diluted POME filtered supplemented with 60 mg/L of urea in 500ml	97	63% of COD	[17]
***Chlorella sorokiniana***	40% diluted POME from anaerobic pond, supplemented with 5% CO2 in 1L flask	90	64.30% of Ammonium 62.30% of Phosphate	[15]
***Chlorella sorokiniana***	40% diluted POME from anaerobic pond, supplemented with 5% CO2 in 1L flask	79.2	75.75% of TN 100% of TP 93.36% of Ammonium 94,50% of Phosphate	[11]
***Chlorella sorokiniana*****CY-1**	30% diluted POME in BG11 medium supplemented with 2.5% of CO2 in 1L flask	150	47.09% of COD 62.07% of TN 30.77% of TP	[34]
***Chlamydomonas sp UKM 6***	16.6% diluted POME from anaerobic pond, supplemented with 2% CO2 in 2L flask	101	12.61% of COD 69.39% of TN 53.33% of TP 100% of Ammonium	[35]

### Effect of algal cultivation on anaerobic pond palm oil mill effluent composition

Microalgae can be used to efficiently uptake inorganic compounds and heavy metals present in wastewater [36]. The presence of nitrogen and phosphorus is a determinant factor favoring eutrophication when the effluent is not properly disposed. On the other hand, these elements are essential for microalgae production [37]. Nitrogen sources such as nitrate, nitrite and ammonia are absorbed by different pathways that converge on the GS-GOGAT cycle for the production of amino acids and ultimately other nitrogenated metabolites [38,39]. Additionally, phosphorus is absorbed as soluble phosphate, in the form of hydrogen phosphate and dihydrogen phosphate, which is further directed to the synthesis of adenosine triphosphate (ATP), nucleic acids, phospholipids and coenzymes [38].

Here, the chemical composition of AP-POME prior and after *Chlamydomonas biconvexa* Embrapa|LBA40 cultivation in airlift flat plate photobioreactors was evaluated. Table 2 shows that there is high organic load (i.e.: Biochemical Oxygen Demand and Chemical Oxygen Demand) and ammonium in AP-POME used for *Chlamydomonas biconvexa* Embrapa|LBA40 cultivation.

**Table 2:** Physicochemical characterization of Anaerobic Pond Palm Oil Mill Effluent (AP-POME) before and after cultivation of *Chlamydomonas biconvexa* Embrapa|LBA40 in airlift flat plate photobioreactors for 5, 10 and 15 days.

Physicochemical characterization	Crude POME	AP-POME	Culture supernatant 5 days	Culture supernatant 10 days	Culture supernatant 15 days
**Biochemical Oxygen Demand (mg.O2/L**^**-1**^**)**	45967.20 (± 3603.27)^e^	20.80 (± 1.70)^a^	38.70 (±0.57)^b^	49.85 (±1.20)^c^	133.95 (±1.63)^d^
**Chemical Oxygen Demand (mg.O2/L**^**-1**^**)**	145890.00 (± 19643.43)^d^	667.00 (± 7.07)^a^	734.00 (±4.24)^b^	768.00 (±11.31)^b^	1272.00 (±28.28)^c^
**Total organic carbon (mg.L**^**-1**^**)**	84073.60 (± 1751.36)	3680.70 (± 243.39)^a^	4678.40 (±778.38)^ab^	4816.00 (±194.60)^ab^	5916.60 (±194.31)^b^
**Nitrate (mg.L**^**-1**^**)**	24.80 (±0.05)^c^	0.72 (±0.00)^b^	0.71 (±0.01)^b^	0.56 (±0.03)^a^	0.47 (±0.04)^a^
**Nitrite (mg.L**^**-1**^**)**	0.45 (±0.05)^b^	0.22 (±0.01)^a^	n.d**	n.d**	n.d**
**Ammonium (mg.L**^**-1**^**)**	95.01 (± 15.03)^b^	44.85 (± 0.64)^a^	n.d**	n.d**	n.d**
**Phosphate (mg.L**^**-1**^**)**	630.00 (± 14.14)^c^	56.75 (±2.47)^b^	1.18 (±0.01) ^a^	1.55 (±0.06)^a^	1.43 (±0.71)^a^
**Potassium (mg.L**^**-1**^**)**	3668.50 (± 338.70)^b^	1850.00 (±70.71)^a^	1575.00 (±106.10)^a^	1750.00 (±70.71)^a^	1875.00 (±35.36)^a^
**pH**	4.59 (± 0.01)^a^	9.17 (± 0.01)^e^	8.37 (±0.02)^c^	8.52 (±0.07)^d^	8.01 (±0.01)^b^

*Results are presented as mean ± error bars of triplicate experiments (n = 3). Means followed by the same letter within a row are not significantly different by One-way ANOVA with Tukey test at the 5% probability level (p≤0.05).

**n.d = not detected.

Additionally, even after the anaerobic stabilization stage, the derived AP-POME still has a residual high biochemical and chemical oxygen demand, as well as a high concentration of nitrogen, that are above the levels permitted by most environmental policies and regulations [10]. Table 2 shows that *Chlamydomonas biconvexa* Embrapa|LBA40 culturing in AP-POME leads to 34.7% reduction of the nitrate after 15 days of cultivation, as well as 99% reduction of the nitrite and ammoniacal nitrogen levels present in the effluent after 5 days of cultivation. Furthermore, phosphate and potassium levels are decreased by 97.9% and 14.8%, respectively, after 5 days of cultivation.

It is noteworthy that, although nitrogen and phosphorous are effectively removed from AP-POME, potassium and TOC, increased in concentration after 10 days of cultivation (Table 2). This observation is parallel with a slight reduction in algal biomass during the same period (Fig 4). Taken together, these findings suggest that the alga reach stationary phase between day 5 and 10, with some cell lysis occuring, potentially from essential nutrient starvation. Therefore, the increase of potassium and TOC detected in culture supernatants is, probably, a consequence of algal cell death and lysis. This hypothesis was further corroborated by the observation of a progressive increase in cell lysis could be observed through microscopical inspection of cultures over time (data not shown).

The use of microalgae for wastewater treatment offers an opportunity for reducing environmental pollution at low costs [36]. Although it has been reported that the cultivation of microalgae in POME can lead to nitrogen and phosphorous compounds reduction levels, algal biomass productivity reported in these previous studies is lower than reported in here (Table 1). Additionally, Table 1 show that the reduction of POME inorganic load was achieved after only 5 days of cultivation. This suggests that the aerobic pond stage, usually used in palm oil industrial plants (Fig 1), could be substituted by high rate photobioreactors, significantly reducing the time and area requirements for wastewater treatment. Such possibility is particularly desirable considering sustainable palm oil production within the Amazon region, since this system could contribute to reduce the pressure for the expansion (or change) of the cultivated area.

### Biomass composition analysis

Medium nutrient recovery translates into the production of algal biomass that could be used to favor the economic viability of the process in a biorefinery strategy (Brasil et al., 2017a). Therefore, algal biomass harvested from *C*. *biconvexa* Embrapa|LBA40 cultures in airlift flat plate photobioreactors was chemically analyzed in order to characterize its potential use as feedstock for added value bioproducts (Table 3).

**Table 3:** Biomass content, yield and compounds of interest accumulated in *Chlamydomonas biconvexa* Embrapa|LBA40 grown in airlift flat plate photobioreactors using Bold Basal medium (BBM) and anaerobic pond palm oil mill effluent (AP-POME).

*Chlamydomonas biconvexa*Embrapa|LBA40	BBM 5 days	BBM 10 days	BBM 15 days	AP-POME 5 days	AP-POME 10 days	AP-POME 15 days
**Biomass productivity (mg.L**^**-1**^**.d**^**-1**^**)**	190.6 (± 15)^a^	147.3 (± 7.5)^b^	89.5 (± 5)^c^	164.7 (± 15)^ab^	156 (± 1.1)^b^	104 (± 17.3)^c^
**Carbohydrate content (%)**	42.53 (± 1.7)^a^	37.17 (± 1.5)^abc^	30.97 (± 2.4)^c^	31.96 (± 3.4)^c^	42.35 (± 4.0)^ab^	34.31 (± 3.8)^bc^
**Carbohydrate productivity (mg.L**^**-1**^**.d**^**-1**^**)**	81.1 (± 3.3)^a^	54.7 (± 2.2)^bc^	27.7 (± 2.2)^d^	52.6 (± 5.6)^c^	66.1 (± 6.3)^b^	35.7 (± 4.0)^d^
**Protein content (%)**	21.6(± 0.12)^b^	18.9 (± 0.14)^c^	17.9 (± 0.08)^d^	26 (± 0.18)^a^	19.3 (± 0.41)^c^	19.0 (± 0.13)^c^
**Protein productivity (mg.L**^**-1**^**.d**^**-1**^**)**	41.1 (± 0.2)^b^	27.8 (± 0.21)^d^	15.7 (± 0.07)^f^	42.8 (± 0.31)^a^	30 (± 0.65)^c^	19.8 (± 0.13)^e^
**Lipids content (%)**	12 (± 0.43)^a^	12.3 (± 0.52)^a^	6.42 (± 0.31)^c^	11.3 (± 0.8)^ab^	9.95 (± 0.45)^b^	6.99 (± 0.60)^c^
**Lipids productivity (mg.L**^**-1**^**.d**^**-1**^**)**	22.9 (± 0.82)^a^	18 (± 0.77)^b^	5.7 (± 2.8)^c^	18.6 (± 1.3)^b^	15.5 (± 0.71)^b^	7.3 (± 0.62)^c^
**Ash content (%)**	6.45 (± 0.56)^a^	4.8 (± 0.04)^b^	5.2 (± 0.12)^b^	6.68 (± 0.2)^a^	4.64 (± 0.08)^bc^	4.06 (± 0.18)^c^

*Results are presented as mean ± standard deviation of triplicate experiments (n = 3). The values with contrast in the table obtained higher content or productivity compared to the other treatments. Means followed by the same letter within a row are not significantly different by One-way ANOVA with Tukey test at the 5% probability level (p≤0.05). AP-POME: Anaerobic pond palm oil mill effluent.

Total carbohydrates comprised the largest fraction detected in the analyzed biomass, reaching 42.53% in BBM and 31.9% in AP-POME cultures after 5 days of algal growth (Table 3). Additionally, *C*. *biconvexa* Embrapa|LBA40 biomass also achieved its highest protein content (26.0%) and lipid content (11.3%) after 5 days of culture in AP-POME (Table 3). Comparatively, *C*. *biconvexa* Embrapa|LBA40 cultivated in sugarcane vinasse achieved 13.5% of carbohydrates, 41.7% of proteins and 1.6% of lipid contents [22]. Indeed, factors like medium composition and cultivation conditions can play a major influence on the biochemical composition of algal biomass [4,15,40].

The contents of lipids and proteins are higher on day 5, both in BBM and AP-POME, decreasing on days 10 and 15 (Table 3). The carbohydrate content also decreases over time in algae biomass derived from BBM cultures. Although there is an increase in the content of carbohydrate in biomass derived from AP-POME on day 10, it falls on day 15. Algae cells have been shown to accumulate carbohydrates or lipids as sources of energy during the stationary phase [41]. The analysis of algae growth kinetics (Fig 4), together with the composition of algae biomass (Table 4), suggests that the stationary growth phase of *C*. *biconvexa* Embrapa|LBA40 in AP-POME occurs before the 10th day of cultivation, followed by a death phase caused by depletion of essential nutrients (ie: nitrogen and phosphorus–Table 2), and characterized by accelerated cell death and decrease in biomass content after day 10. It is worth mentioning that this finding is of practical relevance, since the rate of culture batches/harvest cycles must be optimized to achieve technical-economic viability on an industrial scale. Therefore, the implementation of a fed-batch culture process for *C*. *biconvexa* Embrapa|LBA40 should include harvesting the biomass before the 10th day of growth followed by the renewal of the medium (ie: AP-POME) to replace the depleted nutrients.

**Table 4:** Profile of carbohydrate accumulated in *Chlamydomonas biconvexa*Embrapa|LBA40 grown in airlift flat plate photobioreactors using Bold Basal medium (BBM) and anaerobic pond palm oil mill effluent (AP-POME).

*Chlamydomonas biconvexa* Embrapa|LBA40	BBM 5 days	BBM 10 days	BBM 15 days	AP-POME 5 days	AP-POME 10 days	AP-POME 15 days
Myo-inositol (%)	0.99 (±0,03)^a^	1.18 (±0,02)^a^	1.92 (±0,12)^a^	0.66 (0.05)^a^	1.29 (0.07)^a^	1.46 (0.26)^a^
Arabinose (%)	n.d.	0.52 (±0,03)	n.d.	n.d.	n.d.	n.d.
Galactose (%)	4.04 (±0,01)^a^	2.98 (±0,19)^b^	2.97 (±0,04)^b^	2.93 (0.03)^b^	2.72 (0.14)^b^	2.30 (0.08)^c^
Glucose (%)	77.73 (±0,27)^a^	76.59 (±1,67)^a^	71.48 (±0,45)^b^	77.73 (0.58)^a^	77.13 (0.57)^a^	70.92 (1.90)^b^
Mannose (%)	12.64 (±0,22)^b^	11.82 (±1,37)^b^	18.04 (±0,43)^a^	12.87 (0.04)^b^	11.94 (0.82)^b^	18.82 (1.97)^a^
Ribose (%)	2.22 (±0,13)^a^	2.06 (±0,11)^b^	1.62 (±0,02)^c^	2.16 (0.07)^a^	2.43 (0.07)^a^	2.04 (0.23)^b^
Others (%)	3.44 (±0,14)^c^	3.81 (±0,27)^bc^	4.65 (±0,07)^a^	3.61 (0.09)^b^	4.29 (0.23)^a^	4.04 (0.60)^a^

*Results are presented as mean ± standard deviation of triplicate experiments (n = 3). Means followed by the same letter letter within a row are not significantly different by One-way ANOVA with Tukey test at the 5% probability level (p≤0.05). AP-POME: Anaerobic pond palm oil mill effluent.

Biomolecule profiling is an important step in algal biomass characterization. Carbohydrate profiling revealed the presence of myo-inositol, galactose, mannose, ribose and glucose in *C*. *biconvexa* Embrapa|LBA40 biomass cultivated in AP-POME. Glucose represents the largest fraction corresponding to up to 70% of total carbohydrate (Table 4). Glucose represents the largest fraction corresponding to up to 70% of total carbohydrate (Table 4). It has been reported that algal biomass can be hydrolyzed leading to glucose release, which in turn, can be used for bioethanol and other chemicals production [42]. The second largest fraction observed in *C*. *biconvexa* Embrapa|LBA40 biomass is mannose (Table 4). This is expected since the cell wall from *Chlamydomonas* species is reported to be composed mainly of glycoproteins [43]. Indeed, mannose is the main monosaccharide involved in N-glycosylation and the formation of glycoproteins in *Chlamydomonas reinhardtii* [44,45].

Over the time course of algal culturing (Table 4), it can be observed a slight decrease in the glucose and ribose contents, followed by an increase in the content of mannose. These findings suggest that a reduction in the photosynthetic activity (Calvin–Benson cycle) due to the limitation of nitrogen and phosphorus is occurring after 10 days of algal growth (Table 2) [46,47].

As an important parameter for the quality of the oil, the fatty acid profile changes in algae biomass cultivated under different conditions were analyzed [48]. The most abundant fatty acid in the biomass of *C*. *biconvexa* Embrapa|LBA40 is palmitic acid (ranging from 37% to 42%), followed by oleic acid (ranging from 21% to 28%) (Table 5).

**Table 5:** Profile of Fatty acid methyl esters (FAME) accumulated in *Chlamydomonas biconvexa* Embrapa|LBA40 grown in airlift flat plate photobioreactors using Bold Basal medium (BBM) and anaerobic pond palm oil mill effluent (AP-POME).

*Chlamydomonas biconvexa*Embrapa|LBA40	BBM 5 days	BBM 10 days	BBM 15 days	AP-POME 5 days	AP-POME 10 days	AP-POME 15 days
**Caproic (%)**	2.22 (0.09)^a^	1.87 (0.06)^cd^	1.81 (0.01)^cd^	2.11 (0.12)^ab^	1.73 (0.04)^d^	1.99 (0.09)^bc^
**Palmitic (%)**	37.80 (0.46)^cd^	42.77 (0.65)^a^	40.32 (0.47)^b^	37.55 (0.24)^d^	38.55 (0.31)^cd^	38.90 (0.08)^c^
**Palmitoleic (%)**	2.48 (0.06)^d^	2.51 (0.03)^d^	2.63 (0.04)^c^	2.89 (0.02)^b^	3.08 (0.02)^a^	2.98 (0.03)^b^
**Stearic (%)**	4.14 (0.04)^d^	4.92 (0.09)^b^	4.59 (0.05)^c^	4.42 (0.07)^c^	4.21 (0.05)^d^	5.53 (0.02)^a^
**Oleic (%)**	25.28 (0.48)^c^	28.63 (0.24)^a^	27.40 (0.26)^b^	21.56 (0.20)^d^	25.16 (0.41)^c^	24.53 (0.17)^c^
**Linoleic (%)**	12.99 (0.04)^c^	8.92 (0.26)^e^	12.50 (0.18)^d^	13.91 (0.11)^a^	13.31 (0.05)^bc^	13.46 (0.05)^b^
**Gamma-Linolenic (%)**	0.96 (0.02)^d^	1.90 (0.04)^c^	0.62 (0.01)^e^	2.62 (0.02)^a^	2.13 (0.07)^b^	0.85 (0.01)^d^
**Alpha-Linolenic (%)**	14.09 (0.06)^b^	8.43 (0.28)^e^	10.10 (0.21)^d^	14.92 (0.12)^a^	11.78 (0.08)^c^	11.73 (0.09)^c^

*Results are presented as mean ± standard deviation of triplicate experiments (n = 3). Means followed by the same letter within a row are not significantly different by One-way ANOVA with Tukey test at the 5% probability level (p≤0.05). AP-POME: Anaerobic pond palm oil mill effluent.

Palmitic acid was reported as the largest fraction in *Chlamydomonas sp*. grown in BBM [49] and in other wastewaters, such as in vinasse (32%) and raw chicken manure (35%) [40]. Palmitic acid was also shown to be the main fatty acid produced under sodium acetate stress [50], at nutrient limitation [48,51,52], at high temperatures [53], and at high light intensity [54]. Indeed, a limitation in the content of nitrogen and phosphorous in the AP-POME culture supernatants is observed after 5 days of cultivation (Table 2). Furthermore, *C*. *biconvexa* Embrapa|LBA40 culturing in AP-POME was conducted at high temperatures, reaching 35° C during the light period, and high luminous intensity (i.e.: 35000 lux) (Fig 4). Thus, it seems reasonable to hypothesize that the cultivation conditions observed after 5 days of algal growth might promote an increase in the levels of palmitic acid and of oxygen reactive species [51]. This oxidative stress could also explain the increase in oleic acid (C18:1) levels, and the parallel decrease in the contents of unsaturated fatty acids such gamma-linolenic (C18: 1n9c), linolenic (18: 2n6c) and alpha-Linolenic (18: 2n3), observed over the time course of algal culturing (Table 5) [51]”.

Taken together these results reveal that *C*. *biconvexa* Embrapa|LBA40 biomass could potentially be used as source for the production of biofuels, animal feed, fertilizers, nutraceutical and oleo chemical bio-products [4,12]. In particular, applications of algal biomass as animal feed for in land aquaculture, as soil fertilizers or growth promoters would be of interest in the context of a microalgae production integrated to palm oil industrial plants within the Amazonian region. Indeed, it has been shown that microalgae can be beneficial to aquaculture, as they provide a rich source of micronutrients, lipids and proteins essential for fish farming [55]. Furthermore, different microalgae species can improve soil quality, promote atmospheric nitrogen fixation and produce plant growth hormones [56]. Saadaoui and collaborators [57] have reported a positive effect of applications of algae-based biofertilizer on date palm (*Phoenix dactylifera* L.) cultivation. However, experimentation targeting the evaluation of *C*. *biconvexa* Embrapa|LBA40 biomass for such applications remain an issue to be pursued further.

### Genomic analysis

The Chlamydomonas genus comprise hundreds of flagellated unicellular green algae species. Recently, however, phylogeny studies based on molecular markers have unveiled polyphyletic origin for species originally assigned to Chlamydomonas genus based on morphological data [58,59].

Therefore, a significant reclassification of Chlamydomonas genus species is in course, with several species being reassigned to genera like Oogamochlamys, Chloromonas, Dangeardinia, Ixipapillifera, Rhysamphichloris and Lobochlamys [58,59]. Genomic information about *C*. *biconvexa* species are scarce in the literature and taxonomic assignment of this species has relied basically upon morphological and physiological data [20]. In order to confirm *C*. *biconvexa* Embrapa|LBA40 taxonomic status within Chamydomonas genera and to expand the genetic knowledge basis of this species, mitochondrial DNA (mtDNA) genomic sequencing, annotation and phylogenetic analysis was performed.

**Fig 5:**
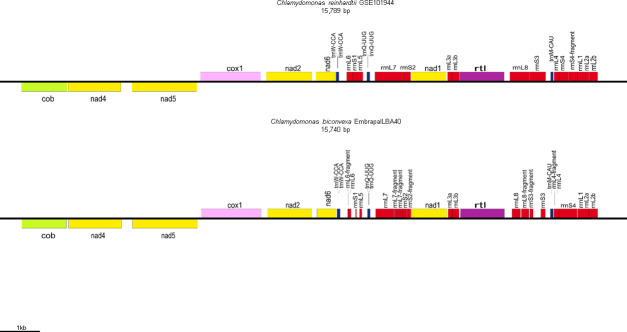
Representation and comparison of the mtDNA from *Chlamydomonas biconvexa*Embrapa|LBA40 strain and mtDNA from the green microalgae reference *Chlamydomonas reinhardtii* [32]. Genes: nad1—Subunit 1 NADH dehydrogenase fragmented; rnL7, rrnL8—Large subunit ribosomal RNA; trnQ-UUG—Transfer RNA, initiation codon amino acid tryptophan; rrnS1, rrnS3, rrnS4—Small subunit ribosomal RNA; trnW-CCA—Transfer RNA, initiation codon amino acid glutamine; nad6—Subunit 6 NADH dehydrogenase; nad2—Subunit 2 NADH dehydrogenase; cox1—Cytochrome c oxidase subunit I; cob—Apocytochrome b protein; nad5—Subunit 5 NADH dehydrogenase; nad4—Subunit 4 NADH dehydrogenase; trnM—Transfer RNA, initiation codon amino acid methionine; nad1—Subunit 1 NADH dehydrogenase.

*C*. *biconvexa* Embrapa|LBA40 mitogenome was assembled through the generation of a consensus sequence with a total of 15980 nucleotides (maximum coverage of 30) and 44.51% of GC content. Fourteen (14) genes could be annotated within the mitogenome, including the protein-coding genes cob, cox1, nad1_0, nad1_1, nad2, nad4, nad5, nad6 and rtl (Fig 5; Table 6). The *C*. *biconvexa* Embrapa|LBA40 mitogenome sequence data was deposited at NCBI GenBank under the accession number MG916975.1.

**Table 6:** Main gene content and features of *Chlamydomonas biconvexa* Embrapa|LBA40 complete mitogenome.

Gene	Strand (+ or -)	Genome position		Length (bp)	Protein product or function
		Start	End		
***nad1_0***	-	0	199	199	Subunit 1 NADH dehydrogenase, fragment
***rrnL***	-	452	1108	657	Large subunit ribosomal RNA
***trnQ (ttg)***	-	1221	1294	73	Transfer RNA. Initiation codon amino acid tryptophan
***rrnS***	-	1572	1602	31	Small subunit ribosomal RNA
***trnW (cca)***	-	1882	1958	75	Transfer RNA. Initiation codon amino acid glutamine
***nad6***	-	2146	2527	382	Subunit 6 NADH dehydrogenase
***nad2***	-	2731	3574	844	Subunit 2 NADH dehydrogenase
***cox1***	-	3813	5316	1504	Cytochrome c oxidase subunit I
***cob***	-	5639	6521	883	Apocytochrome b protein
***nad5***	+	6997	9309	2313	Subunit 5 NADH dehydrogenase
***nad4***	+	9462	10605	1144	Subunit 4 NADH dehydrogenase,
***trnM (cat)***	+	11914	11987	74	Transfer RNA. Initiation codon amino acid methionine
***nad1_1***	+	12975	13731	757	Subunit 1 NADH dehydrogenase, fragment
***rtl***	+	14343	14928	586	Reverse Transcriptase-Like

The tRNAs coding genes encoding trnW, trnQ and trnM, as well rRNAs rrnS and rrnL, the small and large subunit ribosomal RNA, respectively, were also found. The protein-coding genes present in *C*. *biconvexa* Embrapa|LBA40 mitogenome are responsible for cellular respiration, specifically in the oxidative phosphorylation and ATP synthesis pathways, processed in mitochondrion.

Only three tRNAs are coded in mitochondrial genome of *C*. *biconvexa* Embrapa|LBA40, tRNAMet, tRNATrp and tRNAGln (Table 6). The same is observed in the mitochondrial genome of *C*. *reinhardtii* and *C*. *incerta* species, suggesting that there might be importation of other tRNAs to the mitochondrion *in vivo* [60].

On the other hand, the cox2 and cox3 subunit genes, which are characteristic of Reinhardtinia clade species, were not found in *C*. *biconvexa* Embrapa|LBA40 mtDNA. Gene transfer from the mitochondrion to the nucleus throughout the evolution process might explain this feature. According to Pérez-Martínez and collaborators [61], gene transfer process can be considered evolutionary advantageous, since nuclear genes exhibit lower mutation rates, mainly due to a better repair DNA system and genetic stability compared to the mitochondrial environment. Furthermore, the loss of genetic information can also be explained by the need to reduce the energy demand during protein synthesis, leaving only to cox1 subunit the role of cytochrome C oxidase coding inside the mitochondrion.

*Chlamydomonas reinhardtii* is the holotype species of the genus and has been studied for decades as a model for photosynthetic organisms [62]. There is considerable amount of information available about this species genome, mutant strains, culture media, preservation protocols, sexual life cycle and genetic modification methods [4]. Genes like rRNA rrnL and rrnS are fragmented, including three fragments for rrnL and five for rrnS in *C*. *reinhardtii*, a similar gene fragmentation has been reported for the species as *C*. *incerta*, though [60]. tRNAs secondary structures *in silico* predictions based on *C*. *biconvexa* Embrapa|LBA40 mtDNA revealed the presence of both amino acid and anticodon arms, consistent with the cloverleaf shape (S1 Fig). While both trnM and trnQ predicted structures from *C*. *biconvexa* Embrapa|LBA40 possess internal loops between the “T” arm, the *C*. *reinhardtii* trnM is the only gene with this feature (S1 Fig). This feature decreases thermodynamic stability of tRNA structure compared to perfect double-strand pairing [63].

The most conspicuous mitogenomic features reported for species of the Reinhardtinia clade are summarized in Table 7.

**Table 7:** Mitochondrial genome features of Reinhardtinia clade algae.

Genus and species	mtDNA Architecture	Size (kb)	Number of genes	GC Content (%)	GenBank Accession	Reference
***Polytomella sp***.	Linear	13.1	19	42	GU108480.1	(Smith et al. 2010)
***Polytomauvella***	Circular	17.4	19	55	NC_026572.1	(Smith et al. 2013)
***Eudorina sp***.	Circular	20.7	24	Not described	KY442294.1	(Hamaji et al. 2017)
***Volvox carteri***	Linear	29.9	26	44	EU760701.1	(Smith, Lee 2009)
***Pleodorinastarrii***	Circular	20.4	25	38	NC_021108.1	(Smith et al. 2013)
***Chlamydomonas reinhardtii***	Linear	15.8	13	45,2	NC_001638.1	(Vahrenholz et al. 1993)
***C*****.*****biconvexa Embrapa|LBA40***	Linear*	15.7	13	44,5	MG916975.1	This study

*Based on *in silico* analysis.

It can be observed that there is a wide genome architecture variation, including both linear and circular conformations and a wide range of mtDNA sizes, number of genes and GC content. This diversity is characteristic of Chlamydomonadalean algae [64]. It has been proposed that at least three conformational changes (from linear to circular and vice-versa) occurred during evolutionary divergence within this clade. Although, the biological significance of this architecture variation remains elusive, the GC content diversity observed within Chlamydomonadale an algae might be related to ecological adaptation for thermal and/or UV tolerance, as well as specific gene regulation mechanisms [65]. Therefore, Chlamydomonadales mtDNAs can provide a rich source of data to support the reconstruction of phylogenetic history of this group. It is important to highlight, though, that the putatively linear architecture of the mtDNA inferred here base on *in silico* analysis requires future experimental corroboration (e.g.: analysis of telomeres).

**Fig 6:**
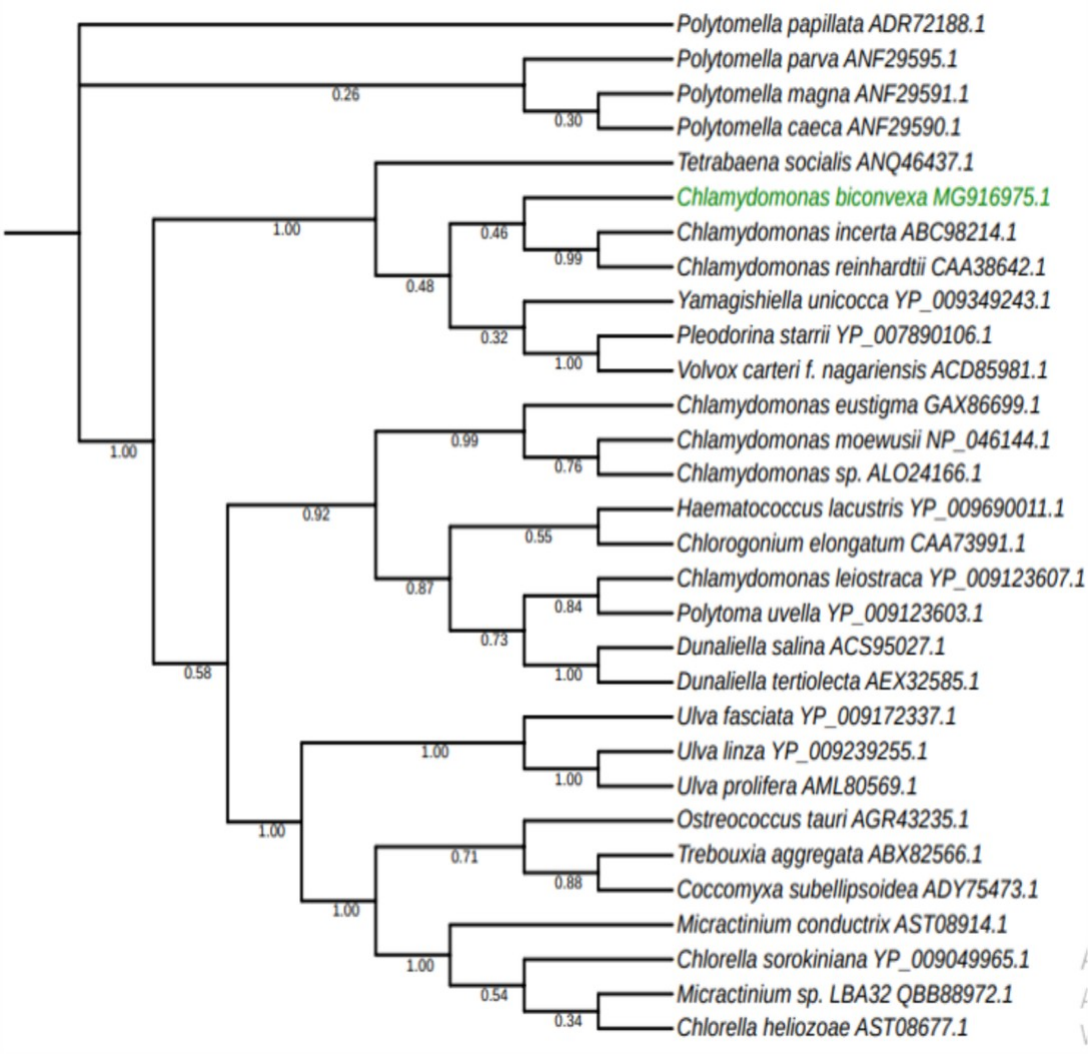
Phylogenetic tree based on mitochondrial cox1 gene sequence inferred by using Maximum Likelihood method and JTT matrix-based model. The bootstrap consensus tree inferred from 100 replicates. Analysis involved amino acid cox 1 gene sequences from 30 microalgae strains and were conducted in MEGA X.

Previous species-level identification based on the analysis of the chloroplast marker, Ribulose Bisphosphate Carboxylase Large subunit gene (*rbcL*), and the nuclear markers, Internal Transcribed Spacers 1 and 2 of the nuclear rDNA (*nu*ITS1 and *nu*ITS2), as well as traditional morphological analysis, have assigned Embrapa|LBA40 to *C*. *biconvexa* species [19]. In order to assess *C*. *biconvexa* Embrapa|LBA40 identification through the use of mitochondrial markers, the cox1 subunit gene from Chlorophyta species was used to reconstruct a phylogenetic tree (Fig 6).

Cytochrome oxidase subunit 1 is a single-copy coding gene informative for phylogenetic analysis in diverse taxa, since it evolves at moderate rates, conserving phylogenetic signatures during the evolutionary process [66]. The tree shows that *C*. *biconvexa* Embrapa|LBA40strain forms a monophyletic branch with *C*. *reinhardtii*, corroborating the taxonomic assignment of *C*. *biconvexa*Embrapa|LBA40 to the Chlamydomonas genus and reinhardtinia clade. This information also suggests that its evolutionary proximity to *C*. *reinhardtii* might allow the interchangeable use of molecular tools and protocols between both species.

## Conclusions

The wastewater tolerant strain *Chlamydomonas biconvex* Embrapa | LBA40 is capable of achieving high productivity when grown in POME, providing biomass that could potentially be used as a source for the production of biofuels, animal feed, fertilizers, nutraceutical or oleochemical bioproducts. The algae culture did not require any supplementation of nutrients other than CO_2_ and was successfully carried out under non-axenic conditions, indicating that *C*. *biconvexa* Embrapa | LBA40 might be a robust strain for industrial scale production. Future studies should focus on scaling up the cultivation and harvesting processes. In addition, the inorganic load of POME is drastically reduced after five days of algae cultivation. This suggests that the aerobic pond stage normally used in industrial palm oil plants could be replaced by high-rate photobioreactors, significantly reducing the time and area required for wastewater treatment. Such a possibility is particularly desirable considering the sustainable production of palm oil in the Amazon region, since this system could contribute to reducing the pressure for the expansion (or change) of the cultivated area. Together, the results unveil a potential use of microalgae in biorefineries integrated with palm oil plants. It is worth mentioning that, once these integrated processes reach industrial scale, the biorefinery may benefit from the commercialization of decarbonization credits provided by the Brazilian National Biofuel Policy (RenovaBio). Therefore, key mechanisms related to technology, economics, environmental sustainability and policy support for algal integrated biorefineries might be available in Brazil in the future. Finally, mitogenomic analysis confirmed that *C*. *biconvexa* Embrapa|LBA40 forms a monophyletic branch with *C*. *reinhardti*, opening up opportunities for the use of molecular tools and protocols developed for this prototypic species in future breeding programs.

## Supporting information

S1 Fig Secondary structure of tRNA present in mtDNA of *Chlamydomonas biconvexa* Embrapa|LBA40 (upper) and *Chlamydomonas reinhardtii* (lower). (DOCX)

S1 Table Molecular identification of microalgae strains based on nuITS2 marker sequence, including the GenBank accession number, closest match species and percentage identity. (DOCX)
