# Supplementary material for: Biochemical and phylogenetic characterization of the wastewater tolerant Chlamydomonas biconvexa Embrapa|LBA40 strain cultivated in palm oil mill effluent
Source: PLoS One. 2021 Apr 7;16(4):e0249089. doi: 10.1371/journal.pone.0249089 (PMC8026047; doi:10.1371/journal.pone.0249089)
Supplement: S1 Fig — (DOCX) [file pone.0249089.s001.docx]

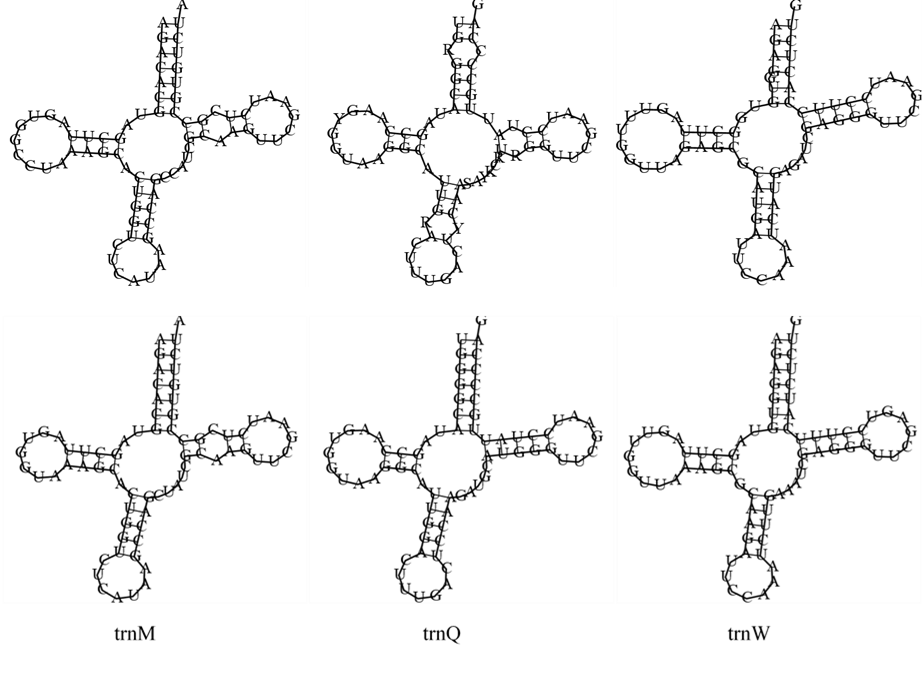


**S1 fig.** Secondary structure of tRNA present in mtDNA of *Chlamydomonas biconvexa* Embrapa|LBA40 (upper) and *Chlamydomonas reinhardtii* (lower).
