# Supplementary material for: Biochemical and phylogenetic characterization of the wastewater tolerant Chlamydomonas biconvexa Embrapa|LBA40 strain cultivated in palm oil mill effluent
Source: PLoS One. 2021 Apr 7;16(4):e0249089. doi: 10.1371/journal.pone.0249089 (PMC8026047; doi:10.1371/journal.pone.0249089)
Supplement: S1 Table — (DOCX) [file pone.0249089.s002.docx]

**S1 table.** Molecular identification of microalgae strains based on nuITS2 marker sequence, including the GenBank accession number, closest match species and percentage identity.

| Strain | GenBank access | Closest match species | Identifity | GenBank access |
| --- | --- | --- | --- | --- |
| *Embrapa*\|LBA8 | KT308046 | *Chlamydomonas* sp. | 97.36% | KY303743 |
| *Embrapa*\|LBA13 | KT308051 | *Coelastrella* sp. | 98.39% | MH176129 |
| *Embrapa*\|LBA32 | KT308068 | *Micractinium* sp. | 99.59% | KM820919 |
| *Embrapa*\|LBA35 | KT308071 | *Desmodesmus* sp. | 99.60% | LR215742 |
| *Embrapa*\|LBA36 | KT308072 | *Desmodesmus* sp. | 100.00% | EU502836 |
| *Embrapa*\|LBA37 | KT308073 | *Chlamydomonas applanata* | 92.34% | MK239987 |
| *Embrapa*\|LBA38 | KT308074 | *Chlamydomonas starrii* | 92.47% | AB983644 |
| *Embrapa*\|LBA39 | KT308075 | *Chlorella sorokiniana* | 100.00% | MK764925 |
| *Embrapa*\|LBA40 | KT308076 | *Chlamydomonas* sp. | 95.06% | MH683945 |
| *Embrapa*\|LBA41 | KT445863 | *Chlamydomonas* sp. | 97.33% | KY303743 |
| *Embrapa*\|LBA45 | KT308081 | *Chloromonas* sp. | 77,98% | MF483442 |
| *Embrapa*\|LBA46 | KT308082 | *Uronema trentonense* | 99.60% | HF920659 |
| *Embrapa*\|LBA47 | KT308083 | *Chlorococcum diplobionticum* | 84.62% | LT594567 |
| *Embrapa*\|LBA48 | MF346373 | *Chlorococcum macrostigmatum* | 93.22% | KX147341 |
| *Embrapa*\|LBA49 | KT308084 | *Chlamydomonas* sp. | 91.13% | MH068693 |
| *Embrapa*\|LBA50 | KT308085 | *Chlorella sorokiniana* | 95.20% | KY229196 |
| *Embrapa*\|LBA51 | KT308086 | *Chlorococcum macrostigmatum* | 93.16% | KX147341 |
